# From Autoimmune Sialadenitis to Central Pain: Hypothesizing Shared Pathogenesis for Fibromyalgia and Primary Sjogren’s Disease and Identifying Essential Screening Strategies

**DOI:** 10.3390/ijms262411821

**Published:** 2025-12-07

**Authors:** Marta Magdalena Jaskólska, Iga Kościńska-Shukla, Kinga Grochowalska, Michał Olech, Zofia Mikołajczak, Magdalena Chylińska, Natalia Aleksandra Dułak, Magdalena Rytlewska, Paulina Pikus, Michał Chmielewski

**Affiliations:** 1 Department of Rheumatology, Clinical Immunology, Geriatrics and Internal Medicine, Medical University of Gdańsk, 80-214 Gdańsk, Polandmichal.chmielewski@gumed.edu.pl (M.C.); 2 Department of Rheumatology, Clinical Immunology, Geriatrics and Internal Medicine, University Clinical Center, Gdańsk, 80-214 Gdańsk, Poland; 3 Division of Quality of Life Research, Medical University of Gdańsk, 80-210 Gdańsk, Poland; 4 Department of Adult Neurology, Medical University of Gdańsk, 80-214 Gdańsk, Poland; 5 Department of Adult Neurology, University Clinical Center, Gdańsk, 80-214 Gdańsk, Poland

**Keywords:** fibromyalgia, Sjogren’s disease, microglia, pain

## Abstract

Even though primary Sjögren disease (pSjD) is mainly associated with sicca symptoms, there are extraglandular manifestations of the disease which affect the quality of life of patients the most and may even be life-threatening. Among the most severe, polyneuropathy and myopathy are worth mentioning. Additionally, clinical observations suggest a higher prevalence of fibromyalgia (FM) in this group of patients, clouding physicians’ assessment and potentially leading to unsuccessful therapeutic decisions. The aim of our study was to evaluate the frequency of pSjD and FM co-occurrence as well as to find the most effective screening tools and markers of such overlap. A total of 97 consecutive patients with diagnosed pSjD were incorporated in the study after obtaining their informed consent. Participants completed a set of broadly available questionnaires, including Fibromyalgia Survey Questionnaire, SF-36 and EULAR Sjögren’s Syndrome Patient-Reported Index (ESSPRI). Data on their laboratory results was collected in the dedicated database. Moreover, patients underwent electroneurographic (ENG) and electromyographic (EMG) testing. Central nervous system (CNS) abnormalities were detected using MRI. Objective disease activity was evaluated based on EULAR Sjögren’s Syndrome Disease Activity Index (ESSDAI). The mean age was 55.3 (range 19.0–78.0 years, SD = 13.9). The disease duration ranged from 2 to 42 years (M = 9.03 years, SD = 7.1 years). Nearly half of the participants (n = 44, 45%) met diagnostic criteria of FM. Interestingly, the diagnosis of FM correlated with CNS involvement. There was no significant correlation between FM and either polyneuropathy/myopathy nor laboratory findings (however, C3c and folic acid concentrations were near the level of significance—mean 1.2 vs. 1.29; *p* = 0.075 and mean 11.35 vs. 9.21; *p* = 0.071, respectively). Within the subcategories of SF-36 and ESSPRI scales, significant positive correlation was noted with ESSPRI total score and ESSPRI pain score (neuropathic subcategory), while a negative correlation was found with SF-36 vitality score, physical functioning score, and the SF-36 total score. FM is common among pSjD patients and should be considered rather a comorbidity requiring different therapeutic approaches. At the fast-paced clinical environment, a concise ESSPRI assessment may be helpful in the initial screening of patients at risk of FM. Even though the origin of this phenomenon is unknown, the concepts of central sensitization and microglia polarization may be potential explanations and more molecular research in this direction could benefit the pSjD patients.

## 1. Introduction

Sjögren disease (SjD) is a rheumatic condition characterized by lymphocytic infiltration in the exocrine glands and parenchymal organs [1], resulting in so called ’sicca symptoms’, which include dryness of the mouth and dryness of the eyes. The above-mentioned signs, along with occurrence of typical antibodies or positive result of labial salivary gland biopsy constitute diagnostic criteria of SjD [2], which may occur as a primary diagnosis or secondary to other rheumatic disorders.

Fibromyalgia (FM) is a chronic condition characterized by widespread pain and constitutes one of the most controversial rheumatic diagnoses, characterized by widespread, nociplastic pain, fatigue, as well as sleep, memory and concentration disturbances [3]. Its prevalence in the general population is estimated at approximately 1–3%. As of now, there is no objective parameter allowing us to diagnose or monitor the development of this condition [4]. The diagnosis is based on the questionnaire completed by the patient, with questions relating to the localization and timing of pain. Even more importantly, despite a severe impact on the quality of life (QoL) and a major economic impact of this condition [5], little is still known about the effectiveness of various therapeutic methods in the management of FM, leading to as many as 90% of patients turning to complementary medicine practices [6].

FM is more common among patients with rheumatic diseases than in the general population. For instance, in the case of vasculitis patients the prevalence of FM reaches 25%, while in spondyloarthritis it may be as high as 53% [7,8]. Hitherto published studies vary widely regarding the frequency of FM in SjD, ranging from 12 to even 50% [8,9,10,11,12,13], likely due to differences in the accepted diagnostic criteria. Even though the connection between conditions had been previously observed and described our understanding of the potential causal connections between these conditions is still limited. Among proposed mechanisms explaining the overlap, three major theories are mentioned: central sensitization, peripheral sensitization, and immune/inflammatory pathways [9]. As of today’s knowledge, FM is immunologically silent. However, some of the researchers attribute the central sensitization to signs of inflammatory activation (cytokine imbalance, lymphocytes activation) and immunization [14]. A retrospective study showed the presence of SjD-associated antibodies in one third of tested FM patients [15]. Unfortunately, FM is still not routinely screened for in the clinical setting, affecting therapeutic effects and patients’ compliance.

Even though complaints such as myalgia are common in FM patients, it is important to differentiate between the widespread, non-inflammatory pain observed in FM patients and clearly defined inflammatory myopathies. The latter are characterized by presence of antibodies, either myositis-specific or myositis-associated, abnormalities within laboratory testing (elevated concentrations of liver enzymes and creatine kinase [CK]) as well as abnormalities in the neurophysiological testing [16]. The diagnosis of FM is more accurate in cases of patients without other diseases, better explaining their complaints (however, it is not a sine-non-quo condition). Nevertheless, a study by Sambataro et al. published in 2023 proves that immunization is not uncommon among FM patients [17].

Despite being mainly recognized for sicca symptoms, SjD carries the risk of concomitant myopathy. Overlap syndrome with myositis (overlap myositis, OM) and inclusion body myositis (IBM) should be considered in every SjD patient who reports muscle weakness or myalgia [16]. The former is usually associated with highly elevated CK (10–15-fold the upper normal limit) while the latter has a more indolent course and frequently begins unilaterally, with less pronounced muscle damage. A specific type of OM is anti-synthetase syndrome. Interestingly, studies show that subclinical myositis in SjD patients is common [18,19,20,21,22,23,24,25] and the clinical symptoms may not be compliant with histopathological examination of the muscles [26].

The aim of this study was to evaluate the frequency of FM among patients with primary Sjögren disease (pSjD) as well as to identify generally accessible and utilized assessment tools potentially useful in detecting patients with FM. For that purpose, a broad range of laboratory tests was analyzed as well as results of patients’ self-assessment tools. Our main focus was drawn to the markers of inflammation, and disease activity evaluation, both subjective and objective. We also put forward a thesis on the potential mechanisms of observed patterns, laying the basis for further research, directed at improvement of patients’ QoL, functional status, and socio-economic outcome.

## 2. Results

The mean age was 55.3 (range 19.0–78.0 years, SD = 13.9). The disease duration ranged from 2 to 42 years (M= 9.03 years, SD = 7.1 years).

Of the 97 participants, 45% (n = 44) concomitantly met the diagnostic criteria of FM. Only one of these patients was male, the remaining 43 cases were represented by female participants.

### 2.1. Primary Endpoints—Objectifying Nervous System Involvement

The first part of our analysis focused on the objective parameters of nervous system involvement, namely abnormalities found in the neurophysiological testing (either in the form of polyneuropathies or myogenic muscle injury) and severity of CNS symptoms and signs, evaluated on the basis of the ESSDAI scale.

Interestingly, individuals with FM had higher ESSDAI CNS scores (M = 0.37, SD = 0.75) than those without FM (M = 0.07, SD = 0.26). A correlation between the CNS involvement evaluated based on the ESSDAI defined criteria and FM diagnosis was significant with *t* test (t statistic = 2.32, *p*-value = 0.025). The overall ESSDAI scale score was not significantly different between the groups based on FM diagnosis (t statistic = 1.68, *p*-value 0.097), with FM-free individuals achieving a mean overall ESSDAI score of M = 1.23 (SD = 1.35), and those with FM, a score of M = 1.73 (SD = 1.55).

Regarding the neurophysiological abnormalities (subtypes of polyneuropathies and myopathy), there was no statistically significant correlation between the diagnosis of FM and their occurrence. Detailed analysis is presented in Table 1.

### 2.2. Secondary Endpoints—Biomarkers and Screening Tools

The FM and non-FM groups were compared in the context of inflammatory parameters, complete blood count, complement system, enzymes, electrolytes, vitamin status, hormones concentration and antibodies. Even though none of the correlations reached statistical significance, it is worth noticing that two factors were close to that—concentration of folic acid (mean 11.35 vs. 9.21; *p* = 0.071) and C3c complement element (mean 1.2 vs. 1.29; *p* = 0.075). The cited *p*-values were not corrected for the multiple comparisons. Nevertheless, these two factors clearly stand out among other incorporated laboratory tests (all the results are included in the Supplementary Material of this article).

Both pSjD and FM severely affected patients’ QoL. We aimed at detecting the aspects of life that would be the most deteriorated by the co-occurrence of pSjD and FM as well as indicating a tool potentially useful for FM screening among pSjD patients. Within the subcategories of SF-36 and ESSPRI scales, significant positive correlation was noted with ESSPRI total score and ESSPRI fatigue (*p* < 0.001) as well as ESSPRI-based neuropathic pain score (*p* = 0.003), while a negative correlation was found with SF-36 vitality score, physical functioning score, and the SF-36 total score. Detailed analysis of the latter scale is presented in Table 2.

**Figure 1:**
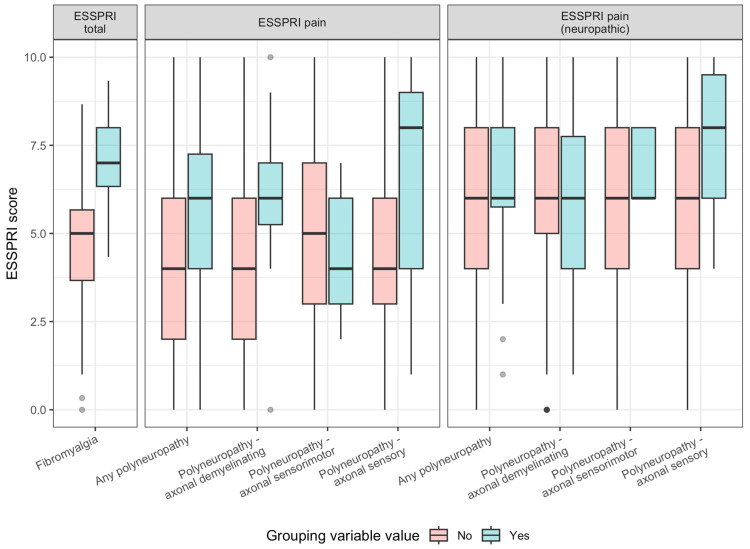
Test effect for concomitant diagnosis of polyneuropathies/fibromyalgia and ESSPRI scores.

Among the clinical scales, ESSPRI total score proved to be a useful tool in detecting patients with concomitant FM while maintaining satisfactory group sizes. The difference in results was striking, as presented in Figure 1. All the correlations for ESSPRI scores are detailed in Table 3.

**Figure 2:**
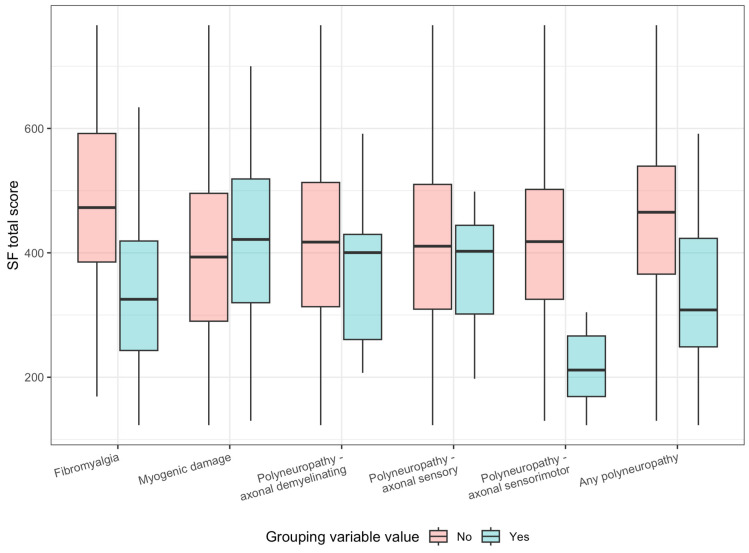
Effect size for SF-36 total score in correlation with polyneuropathy or FM diagnosis.

Similarly, SF-36 total score showed strong correlation with the co-occurrence of FM. Due to the limited number of patients diagnosed with various polyneuropathy subtypes inference was not possible. When considering the SF-36 total score vs. concomitant diagnosis of any of the polyneuropathy subtypes, we obtained groups of 60 vs. 26 patients with moderate power as a result. The calculations are presented in Table 4 and Figure 2.

No statistically significant correlation was found between the concomitance of FM with pSjD and age, disease duration, or stressful life events measured with the H-R scale.

## 3. Discussion

Results obtained in this study comply with the view of high prevalence of FM among the pSjD patients. As of today, this phenomenon is not well understood. Research in the field of biochemical abnormalities observed in SjD suggests several hypotheses explaining the frequency of neuropsychological symptoms.

SjD is generally associated with the dryness of oral cavity and eyes. However, extraglandular manifestations may be equally common and lead to severe deterioration of patients’ condition and QoL. One of the frequently overlooked manifestations is the involvement of the nervous system, affecting both the peripheral nervous system (PNS) and CNS. Studies within this area are highly heterogenous in terms of definitions and methods, leading to discrepancies in the results. The reported prevalence of neuropathy in this group of patients ranges from 8 to 49% [27,28,29,30,31]. In the available literature, several pathomechanisms are suggested. One of the relatively well-described includes kynurenine (KN) metabolic pathway, stimulated by the elevated concentrations of interferon γ (IFN γ) [32]. Impaired tryptophan metabolism has been recognized as one of the major factors in the development of several psychiatric conditions. Recent research sheds light on the importance of dysfunctional enzymes, tryptophan-2,3-dioxygenase (TDO) and interferon responsive indoleamine 2,3-dioxygenase (IDO1), and subsequent shift from serotoninergic pathway to kynurenic pathway of tryptophan catabolism in the dysregulation of immune system [33] as well as SjD onset and course, particularly in terms of neuropsychiatric symptoms [34,35,36,37]. According to the study by Li et al. in a group of 115 SjD patients, a specific defect in the 2,5-oligoadenylate synthetase 1 (OAS1) led to resistance to IFN γ, hence leading to elevated concentrations of this cytokine and consequently increased risk of lymphoma, neuropathy and severe fatigue [38]. Most of the clinicians managing SjD cases share the observation of highly diverse phenotypes in this disease—while some of the patients suffer mainly from classic sicca symptoms, in others positive SSA or SSB antibodies appear to be almost accidental findings in the diagnostic process of new onset seizures, headaches or polyneuropathy. The impact of kynurenine on several areas of the brain, including hippocampus, constitutes a plausible explanation for this clinical observation [33].

In our view, one of the most interesting and significant findings of the study is the positive correlation between CNS involvement in the course of pSjD and concomitant diagnosis of FM. Numerous hypotheses associated with CNS involvement in pSjD are controversial and doubtful, mainly due to the immunologically privileged character of the CNS with the blood–brain barrier guarding it from infiltration of antibodies, toxins and pathogens. Even though its function may become impaired secondary to generalized inflammation, the severity of such impact remains uncertain in otherwise healthy adults. Nowadays, one of the most convincing concepts behind described phenomena are ‘central sensitization’ and ‘microglia inflammation’.

Even though the term ‘central sensitization’ was first used as late back as 1989 [39], its understanding is still limited. It originates from observations conducted on rats in which neurons of the spinal cord were becoming hyperexcitable even over extended time after the injury. Such a concept clarifies the ideas of hyperalgesia (excessive reaction to pain stimulus), allodynia (response with pain to unpainful stimulus), and global sensory hyperresponsiveness (sensitivity to both internal and external stimuli).

However, this approach justifies only excessive response to objectively present stimulus, whether painful or not. Yet the main characteristic feature of FM is the experience of pain even in the absence of any potentially harmful factors. Such a phenomenon can be explained with the concept of nociplastic pain. The term was coined in 2016 to fill in the gap between nociceptive and neuropathic pain, in order to better understand and manage patients suffering long-term with no evident stimulation nor nerve damage [40].

Microglia are the phagocytes associated with the brain, present mainly in the resting condition. Depending on the character and timing of the noxious stimulus, microglia transform into one of the active forms in the broad spectrum of activation pathways. The classical pathway, proinflammatory in nature, is described as M1. Alternatively activated microglia, M2, is anti-inflammatory in nature. The M1 activation is mainly evoked by lipopolysaccharide (LPS), IFN γ, tumor necrosis factor α (TNFα) and trauma-induced cellular debris; such activation leads to production of among others TNFα, interleukin 1β (IL-1β), interleukin 6 (IL-6) and inducible nitric oxide synthase (iNOS) as well as expression of CD86, CD14, CD16, CD32 and CD42 [3]. On the other hand, M2 activation is associated with cytokines such as IL-4, IL-10 and IL-13, neurotrophic mediators, colony-stimulating factor 1 (CSF-1) as well as expression of CD163 and CD206. The imbalance between the two pathways is one of the proposed factors in the development of neuropathic pain, regardless of the underlying cause [41,42,43]. Similarly, the disturbance of natural M1/M2 ratio has been suggested as one of the pathomechanisms involved in the nociplastic pain development, including fibromyalgia [3]. Moreover, based on animal studies, some authors even suggest targeting the disturbed polarization process as a therapeutic strategy [44,45]. In our study group a positive correlation was observed between CNS involvement in the course of pSjD and concomitant diagnosis of FM. The microglia polarization could be therefore implicated in both processes, constituting a noteworthy diagnostic and therapeutic target. As of now, there are no clear indications whether M1/M2 markers should be evaluated in the serum or cerebrospinal fluid. Assessment of the latter would carry the necessity of hospitalization and potentially frequent repetition of high-risk procedures, often poorly tolerated by the patients. Nevertheless, considering the impact of FM on patients’ QoL and their economic status, further research into the subject would be highly desired and socially beneficial. The majority of available research, including the hereby presented, focuses on the prevalence of FM among patients with other rheumatic diseases. However, a bidirectional connection between the conditions cannot be excluded, as in the analysis performed by Gau et al. [14]. A theory that it is the FM which leads to chronic inflammation and predisposes individuals to development of connective tissue disorders is comparably plausible.

Organization of healthcare systems worldwide varies broadly, oftentimes preventing specialists from regular assessment of patients’ well-being or referring them to a broader laboratory work-up—whether due to financial shortages or insufficient time dedicated to appointments. Our findings prove that neither patients with SD nor FM present typically expected abnormalities in the laboratory testing, that is elevated concentrations of inflammatory markers or anomalies in the CBC. No correlation was found between the presence of antibodies and co-occurrence of the two diagnoses, indicating a novel, non-immune mechanism. These results stand to some extent in opposition to the study published by Applbaum et al. [15] who noted higher prevalence of novel, early-stage antibodies among FM patients who later developed pSjD. Even though it is definitely an interesting direction for future research, our objective was to identify a broadly accessible tool, preferably already frequently utilized in daily practice. With folic acid being an important factor of significant metabolic tracts and with a known role of C3c in the course of autoimmune diseases, particularly those of neurological nature, it is justified to expect metabolic disturbances in the CNS being involved in the development of FM, which pathogenesis has not, as of today, been explained at the molecular level.

ESSPRI and SF-36 are broadly recognized and utilized tools for evaluating patients’ QoL. In rheumatologists’ daily practice they are mainly associated with the disease activity, and unfavorable results direct physicians towards intensification of immunosuppressive treatment or symptomatic management of reported complaints. Our findings suggest that unsatisfactory results of the therapy, expressed predominantly as elevated scores in ESSPRI (total score and pain with dominance of neuropathic complaints) and diminished values of SF-36, particularly within the ‘vitality’ component, should direct clinicians toward the pursuit of other, seemingly unrelated problems, such as concomitant diagnosis of FM. Even though, as of today, the successful management strategy of FM remains unclear and understudied, the clinical gold standard includes antidepressive medications (pregabalin, duloxetine, tricyclic antidepressants) and mindfulness techniques. Considering that FM diagnosis is based on self-reported complaints, it seems justified to incorporate the routine screening test of FM in case of patients with disadvantageous self-assessment in ESSPRI and SF-36 scales. The clinical value of this observation lies in the fact that particularly ESSPRI, with its concise form and ease of both completion and analysis, should be considered as an initial screening tool in the busy environment of out-patient clinics, before offering a more extensive and time-consuming full FM diagnostic questionnaire. Notably, our findings related to ESSPRI and ESSDAI are compliant with previously published study [9].

Stress is considered an important factor in the onset and course of both fibromyalgia as well as broadly understood autoimmune diseases. In our study, however, more stressful life events measured in the H-R scale did not carry an increased risk of FM diagnosis among pSjD patients. Nevertheless, its impact on the development of depression and subsequent sensitization should always be considered, particularly among female pSjD patients, who were proven more prone to neuropsychiatric manifestations of pSjD [13]. In the case of our study, the gender distribution was 11:1 with dominance of females, resulting in large difference in the sample sizes and preventing us from comparison of these aspects between sexes.

Even though the treatment of FM becomes an increasingly studied subject, there is still a shortage of large, randomized trials within the field. The majority of studies focus on lifestyle modifications and mindfulness techniques, with pharmacotherapy being largely underestimated. Interest in the use of lidocaine in cases of neuropathic pain has been on the rise for years [46]. Moreover, trials on cancer cells, SARS-CoV-2 patients, and in perisurgical management give promising results on the impact of lidocaine on the pro- and anti-inflammatory cytokines balance restoration, affecting, among others, the above-mentioned IFN γ [47,48,49,50]. Combined with the data on the anti-inflammatory action of local anesthetics associated with the inhibition of ion exchange, lidocaine became a substance of interest in the management of FM resistant to typical treatment. The design of studies varies widely, leaving clinicians with a broad range of dosages and therapeutic schemes to be implemented [51]. Doses between 2 and 7.5 mg per infusion are generally considered safe, but should always be administered as an in-patient procedure, due to the risk of arrythmia. The authors’ own experience complies with that presented by Wilderman et al. [52], with the effect of infusions being delayed, oftentimes experienced as late as after 6–8 infusions performed in monthly intervals. Therefore, patient–physician communication becomes of the utmost importance. If patients are not prepared for a prolonged anticipation of results, they are likely to discontinue the therapy. Bearing in mind the previously described mechanisms of M1/M2 polarization of microglia, it seems probable that the gradual anti-inflammatory action of lidocaine at the CNS level is in fact the essence of its action.

Some of the medications suggested in the literature as potentially affecting the above-described imbalance in the M1/M2 microglia polarization include minocycline, naltrexone, infliximab and dextromethorphan. Although as of now, none of them is routinely used in FM management, future results from larger clinical trials could bring significant improvement to the perception and treatment of this understudied condition.

## 4. Materials and Methods

### 4.1. Ethical Committee Approval

This study constitutes a part of a major project, which obtained the local Ethical Committee Approval no KB/438/2024 granted on the 13 September 2024.

### 4.2. Study Group

A group of 97 consecutive patients diagnosed with pSjD were included in the study. Considering the prevalence of pSjD described in the literature (0.5–5%), maintaining the confidence level of 95% and margin error of 5%, accounting for 38,000,000 citizens of Poland, a representative sample size ranges from 16 to 73 individuals. The gender composition of the group was compliant with the literature data on pSjD prevalence and established at 89 women and 8 men (ratio 11:1).

### 4.3. Inclusion and Exclusion Criteria

Inclusion criteria:

- age at least 18 years old
- informed consent
- pSjD diagnosis based on the ACR-EULAR 2016 Classification Criteria

Exclusion criteria:

- age below 18 years old
- lack of informed consent
- diagnosis of secondary Sjögren syndrome
- other systemic diseases, uncontrolled or in the periods of exacerbation, leading to impaired pain perception such as hypo/hyperthyroidism, diabetes mellitus, dyselectrolytemia, severe vitamin D and B12 deficiencies, anemia, active infections.

### 4.4. Evaluation and Data Collection

Data on patients’ age, gender and disease duration were collected based on their medical charts accessible in the internal IT system.

(1) Questionnaire

Everyone who consented to participation in the study completed a questionnaire, constituting the following scales:

- EULAR Sjögren Syndrome Patient Reported Index (ESSPRI) [53] extended with specifying the type of experienced pain (neuropathic, articular, muscular)
- Holmes-Rahe Life Stress Inventory (H-R) [54]
- Short Form Health Survey (SF-36) [55]
- American College of Rheumatology revised 2016 diagnostic criteria for fibromyalgia [56].

(2) Disease Activity

All patients were evaluated by an experienced rheumatologist, specializing in the management of SjD patients. The presence and severity of organ involvement was assessed based on the EULAR Sjögren Syndrome Disease Activity Index (ESSDAI) [57].

(3) Neurophysiological Tests

All patients reporting symptoms of peripheral neuropathy were referred for neurophysiological testing, performed by an experienced neurologist. Electrophysiological evaluation included nerve conduction study (NCS) performed on a Dantec Keypoint G4 EMG/NCS/EP Workstation (Natus, Middleton, WI, USA) in a standardized environment (temperature of 32–34 °C). Studies were performed under the protocol recommended by the American Association of Neuromuscular and Electrodiagnostic Medicine (AANEM) [58]. NCS were performed in the following nerves:

1. Motor fibers: the median nerve (compound muscle action potential (CMAP) recorded from the Abductor Pollicis Brevis muscle), the ulnar nerve (CMAP recorded from the Abductor Digiti Minimi muscle), the radial nerve (CMAP recorded from the Extensor Indicis Proprius muscle), and the peroneal nerve (CMAP recorded from the Extensor Digitorum Brevis muscle).

2. Sensory fibers: the median nerve using an orthodromic technique (sensory nerve action potentials (SNAP) stimulation digits I and III, recording wrist), the ulnar nerve using an orthodromic technique (SNAP stimulation digit V, recording wrist), the radial nerve using an antidromic technique (stimulation forearm, recording an anatomical snuffbox), and the sural nerve using an antidromic technique (lower leg to the lateral malleolus). A detailed description of the examined nerves, along with their respective reference ranges, is provided in Table 5. Polyneuropathies were classified following the European Standardized Telematic Tool to Evaluate Electrodiagnostic Methods (ESTEEM) guidelines [59]. Classification included axonal, demyelinating, or mixed polyneuropathies. Observed abnormalities were categorized as one of the following: carpal tunnel syndrome (CTS), demyelinating or axonal- demyelinating, mononeuropathy, multiple mononeuropathy (subcategorized as moto-sensory, motor or sensory), polyneuropathy (demyelinating, axonal moto-sensory, axonal sensory, axonal motor or axonal demyelinating) or radiculopathy. Abnormalities within the CNS were detected using magnetic resonance imaging (MRI).

Electromyography (EMG) studies were performed using a Dantec Keypoint G4 EMG/NCS/EP Workstation, following the protocol recommended by the American Association of Neuromuscular and Electrodiagnostic Medicine [58,60], and conducted by a certified electromyographer (MC). Concentric needle EMG (9013S0032 Dantec^®^ DCN Disposable Concentric Needle Electrode, Galway, Ireland) was performed bilaterally in the deltoid (DELT) muscles and unilaterally in the biceps brachii (BB) and vastus lateralis (VL) muscles. Quantitative motor unit action potential (MUAP) analysis included assessment of amplitude, duration, size index, polyphasic percentage, and interference pattern (IF). A proportion of polyphasic MUAPs exceeding 20% was considered indicative of an abnormal finding. Early recruitment observed during interference pattern analysis supported the diagnosis of a myopathic disorder. A minimum of 20 MUAPs was recorded for each muscle. Reference values are presented in Table 6. EMG abnormalities were classified as follows:

1. Myopathic—needle EMG demonstrated short-duration, low-amplitude, polyphasic MUAPs with a reduced size index, typically accompanied by an interference pattern and early recruitment in weak muscles. The findings were classified as active myopathic when abnormal spontaneous activity was present, including fibrillation potentials and positive sharp waves.

2. Neurogenic pattern—MUAPs were characterized by increased amplitude, prolonged duration, and elevated size index, with or without polyphasia, typically accompanied by reduced recruitment. The findings were classified as active neurogenic when abnormal spontaneous activity was present, including fibrillation potentials and positive sharp waves.

(4) Laboratory Findings

Moreover, extensive laboratory results of the participants were collected. The tests included both basic work-up, such as complete blood count (CBC), markers of the liver and renal function, pituitary-thyroid axis hormones, electrolytes and vitamins levels, iron deficiency indices, inflammatory markers (C-reactive protein (CRP), erythrocytes sedimentation rate (ESR), fibrinogen), as well as immunological testing—antinuclear antibodies (ANA), immunoblot, complement C3 and C4 elements, rheumatoid factor (RF), anti-cyclic citrullinated peptide antibodies (anti-CCP), beta2-microglobulin (B2MG), serum protein electrophoresis.

(5) Study Design

Depending on the result of American College of Rheumatology revised 2016 diagnostic criteria for fibromyalgia, participants were divided into two groups: those with concomitant diagnosis of pSjD and FM and those who were FM-free. All of the above-mentioned parameters were compared between the groups in order to determine clinically useful screening tools as well as to establish any potential correlation between disease activity or organ involvement and diagnosis of FM.

### 4.5. Data Analysis

Validation analyses were performed using the R language (4.5.1) and RStudio software (2025.09.2+418). The analyses also utilized the tidyverse (2.0.0) [61] and rstatix (0.7.2) [62] packages. Correlation between the occurrence of FM and various forms of polyneuropathy as well as myopathy was checked with χ^2^ test (even though some of the variables were characterized by small counts, due to the general, large number of observations within the group it was decided not to replace the χ^2^ test with the Fisher test).

The correlation between CNS involvement (measured predominantly as defined in ESSDAI guidelines) and FM occurrence was evaluated with the use of *t* test potential skewness in the sample distributions is mitigated by the effects of the Central Limit Theorem and the sample sizes. Another factor contributing to the choice of strategy was the character of the variable describing the CNS involvement, which is a comparative scale.

Furthermore, laboratory findings were evaluated in the context of concomitant FM. For this purpose, the *t*-test was performed. For the evaluation of the power of the effect d Cohen factor was used.

Since our dependent variable (presence of FM diagnosis) is dichotomous and the well-being diagnosis was performed using the multidimensional SF-36 questionnaire, two parallel approaches were employed. For the detailed SF-36 scale scores, backward logistic regression was used to investigate which variables significantly explained the variance of the FM variable. Separately, analyses were conducted for the SF-36 total score: because this score is highly correlated with the detailed SF-36 scale scores, it was decided to use a *t*-test to check whether the variable differs between groups, including those defined by the FM variable.

## 5. Conclusions

Significantly worse results in the self-assessment tools, SF-36 and ESSPRI, among patients with co-occurring FM and pSjD prove the impact of these diagnoses on the QoL. Each of these conditions requires a different therapeutic approach, therefore the physicians’ failure to identify the major underlying problem may lead to unsatisfactory results and consequently diminish patients’ trust and decrease compliance. Unlike other rheumatic conditions, patients with FM and pSjD tend to deteriorate without major abnormalities within known laboratory tests, which also proves right in the presented study. Despite extensive laboratory assessment, there were no significant differences among the results. Therefore, no objective laboratory marker for early detection of increased FM risk may be suggested. Based on our findings, we recommend the routine use of ESSPRI and SF-36 scales not only for evaluation of disease activity, but also as a screening tool for early detection of FM. The positive correlation of CNS involvement in pSjD with the diagnosis of FM constitutes a basis for further search of common pathomechanisms at a neuronal level, with potential shift in our understanding and classification of these conditions.

## Figures and Tables

**Table 1:** Correlation between diagnosis of polyneuropathies/myogenic muscle injury and co-existence of FM and pSjD.

ENG/EMG Abnormality	Fibromyalgia Co-Occurrence				χ^2^ Test		
	No/No	No/Yes	Yes/No	Yes/Yes	Statistic	df	*p*-Value
polyneuropathy							
axonal-demyelinating	41	35	7	7	<0.01	1	1.00
axonal-sensory	44	39	4	3	<0.01	1	1.00
axonal-sensorimotor	46	39	2	3	0.02	1	0.88
demyelinating	24	12	0	2	1.32	1	0.25
any polyneuropathy	35	27	13	15	0.43	1	0.51
myopathy	11	15	39	27	1.50	1	0.22

No/No—free from ENG/EMG abnormalities and FM; No/Yes no ENG/EMG abnormalities with diagnosed FM; Yes/No ENG/EMG abnormalities in patients without FM; Yes/Yes ENG/EMG abnormalities in FM patients.

**Table 2:** Coefficients of logistic models for FM as dependent variable and SF-36 as independent variables at the beginning and the end of backward regression procedure.

SF-36 Score	Full Model			Reduced Model		
	Odds Ratio	95% CI	*p*-Value	Odds Ratio	95% CI	*p*-Value
(Intercept)	31.13	(2.49, 518.29)	0.010	54.66	(9.15, 471.17)	<0.001
vitality	0.96	(0.93, 0.99)	0.022	0.96	(0.94, 0.99)	0.008
role-physical	0.97	(0.94, 0.99)	0.021	0.96	(0.93, 0.98)	0.000
bodily pain	1.00	(0.98, 1.03)	0.915			
general health	1.00	(0.95, 1.06)	0.851			
physical functioning	0.99	(0.97, 1.00)	0.165			
emotional functioning	1.00	(0.98, 1.01)	0.781			
social functioning	0.98	(0.96, 1.01)	0.257			
mental health	1.02	(0.98, 1.07)	0.304			
Nagelkerke R^2^	0.41			0.36		

**Table 3:** Correlation between ESSPRI scores and co-occurrence of neuropathy and fibromyalgia.

ESSPRI Score/Co-Morbidity	IV = 0			IV = 1			*t* Test		
	N	M	SD	N	M	SD	Stat.	df	*p*-Value
**ESSPRI pain score**									
polyneuropathy axonal-demyelinating	75	5.93	2.66	14	5.71	2.73	0.28	17.9	0.79
polyneuropathy axonal-sensory	82	5.76	2.65	7	7.57	2.30	−1.98	7.4	0.09
polyneuropathy axonal-sensorimotor	84	5.85	2.72	5	6.80	1.10	−1.67	7.4	0.14
any polyneuropathy subtype	61	5.67	2.77	28	6.39	2.36	−1.26	61.0	0.21
**ESSPRI based neuropathic pain score**									
polyneuropathy axonal-demyelinating	74	4.45	2.91	14	6.07	2.43	−2.22	20.7	0.04
polyneuropathy axonal-sensory	81	4.56	2.79	7	6.43	3.64	−1.33	6.62	0.23
polyneuropathy axonal sensorimotor	83	4.72	2.94	5	4.40	2.07	0.33	5.02	0.76
any polyneuropathy subtype	60	4.20	2.87	28	5.79	2.66	−2.54	56.7	0.01
**ESSPRI total score**									
fibromyalgia	53	4.67	1.99	43	7.09	1.31	−7.15	90.4	<0.01

IV: independent variable; N: number; M: mean; SD: standard deviation.

**Table 4:** SF-36 total score in correlation with concomitant polyneuropathy or FM diagnosis.

Co-Morbidity	No			Yes			*t* Test			Effect	
	N	M	SD	N	M	SD	Stat.	df	*p*-Value	Cohen’s d	Size
Fibromyalgia	53	476.59	132.94	44	336.74	119.57	5.33	89.4	<0.001	1.11	Large
Myopathy	26	397.87	158.65	62	424.03	141.13	−0.73	42.7	0.471	−0.17	Negligible
Polyneuropathy axonal-demyelinating	74	420.95	150.10	12	367.86	119.38	1.37	17.2	0.187	0.39	Small
Polyneuropathy axonal-sensory	79	417.39	149.66	7	370.04	107.04	1.08	8.2	0.312	0.36	Small
Polyneuropathy axonal sensorimotor	81	425.80	141.41	5	214.86	72.86	5.83	6.06	0.001	1.88	Large
Any polyneuropathy subtype	60	445.41	147.02	26	339.99	118.95	3.50	58.24	<0.001	0.79	Moderate

N: number; M: mean; SD: standard deviation.

**Table 5:** The table summarizes the characteristics of the examined nerves together with the electrophysiological laboratory reference values established for the assessed Sjögren population.

Nerve	Amplitude LLN	Distal Latency ULN [Distance]	Conduction Velocity LLN
Motor nerve conduction study			
Median (APB)	5 mV	4.2 ms [80 mm]	50 m/s
Radial (EIP)	3.5 mV	3.5 ms [120 mm]	51 m/s
Ulnar (ADM)	7 mV	3.2 ms [80 mm]	50 m/s
Peroneal (EDB)	3 mV	4.5 ms [80 mm]	40 m/s
Sensory nerve conduction study orthodromic			
Median dig I	10 µV	3.5 ms [80 mm]	49 m/s
Median dig III	10 µV	3.4 ms [80 mm]	50 m/s
Ulnar dig. V	8 µV	3.5 ms [80 mm]	50 m/s
Sensory nerve conduction study antidromic			
Radial	10 µV	3.7 ms [100 mm]	51 m/s
Sural	9 µV	4.5 ms [100 mm]	40 m/s

Abbreviations: LLN, Lower Limit of Normal; ULN, Upper Limit of Normal; APB, Abductor Pollicis Brevis muscle; EIP, Extensor Indicis Proprius muscle, ADM, Abductor Digiti Minimi muscle, EDB, Extensor Digitorum Brevis muscle; dig, digit; mV, millivolt; ms, milliseconds; mm, millimeter; µV, microvolt; m/s, meters per second.

**Table 6:** A comprehensive overview of the reference values for motor unit action potentials parameters in the examined muscles of patients with Sjögren’s disease.

Muscle	Amplitude [µV]	Duration [ms]	Size Index
Deltoid	540	10.2	0.84
LLN-ULN	450–630	9.0–12.0	0.53–1.15
Biceps brachii	450	10.0	0.68
LLN-ULN	390–560	8.5–11.5	0.34–0.99
Vastus lateralis	700	11.9	1.28
LLN-ULN	490–980	9.9–13.9	0.9–1.68

Abbreviations: LLN, Lower Limit of Normal; ULN, Upper Limit of Normal; µV, microvolt; ms, milliseconds.

## Data Availability

The original contributions presented in this study are included in the article/Supplementary Materials. Further inquiries can be directed to the corresponding author.

## Supplementary Materials

The following supporting information can be downloaded at: https://www.mdpi.com/article/10.3390/ijms262411821/s1.
