# Supplementary material for: From Autoimmune Sialadenitis to Central Pain: Hypothesizing Shared Pathogenesis for Fibromyalgia and Primary Sjogren’s Disease and Identifying Essential Screening Strategies
Source: Int J Mol Sci. 2025 Dec 7;26(24):11821. doi: 10.3390/ijms262411821 (PMC12733063; doi:10.3390/ijms262411821)
Supplement: Supplementary file 1 [file ijms-26-11821-s001.zip › ijms-4009033-supplementary.pdf]

variable	non-FM			FM			t test			Cohen's d	
	N	M	SD	N	M	SD	statistic	df	p-value	effect	magnitude
hemoglobin	53	13.50	1.35	44	13.19	1.30	1.15	92.9	0.255	0.233	small
leukocytes	53	5.34	2.03	44	5.99	2.67	-1.32	79.2	0.191	-0.272	small
lymphocytes	53	1.59	0.67	44	1.67	0.77	-0.55	86.3	0.586	-0.112	negligible
neutrocytes	53	3.12	1.58	44	3.71	2.42	-1.40	71.5	0.165	-0.291	small
platelets	53	244.17	52.58	44	248.91	50.38	-0.45	93.0	0.652	-0.092	negligible
CRP	53	2.03	3.33	44	1.91	3.27	0.18	92.3	0.859	0.036	negligible
ESR	53	15.51	17.70	44	14.14	10.96	0.47	88.3	0.642	0.093	negligible
creatinine	53	0.81	0.22	44	0.82	0.21	-0.23	93.4	0.822	-0.046	negligible
GFR	53	82.92	11.17	44	79.25	15.76	1.30	75.5	0.198	0.269	small
AspAT	53	26.11	6.96	44	26.66	7.41	-0.37	89.4	0.711	-0.076	negligible
AlAT	53	22.30	11.31	44	25.80	13.24	-1.38	85.1	0.171	-0.284	small
GGTP	53	26.55	32.93	44	32.02	35.09	-0.79	89.4	0.434	-0.161	negligible
fibrinogen	52	3.44	0.76	44	3.46	0.63	-0.11	94.0	0.910	-0.023	negligible
iron	53	92.84	33.53	44	99.45	38.99	-0.89	85.4	0.378	-0.182	negligible
ferritin	53	88.06	88.32	44	74.22	61.44	0.91	92.3	0.367	0.182	negligible
vitamin B12	53	455.79	210.71	44	490.80	215.13	-0.81	91.0	0.423	-0.164	negligible
vitamin D	53	44.92	17.01	44	44.20	14.32	0.22	95.0	0.823	0.045	negligible
sodium	53	135.12	26.17	44	136.91	20.62	-0.38	94.8	0.706	-0.076	negligible
potassium	53	9.34	26.23	44	7.35	20.47	0.42	94.7	0.675	0.085	negligible
phosphates	53	3.40	0.49	43	3.31	0.50	0.93	89.2	0.353	0.192	negligible
magnesium	53	2.02	0.16	44	2.03	0.15	-0.54	93.4	0.590	-0.110	negligible
C3c complement component	49	1.20	0.16	40	1.29	0.26	-1.81	63.2	0.075	-0.394	small
C4 complement component	49	0.22	0.07	40	0.22	0.07	0.01	79.4	0.990	0.003	negligible
gammaglobulins	53	15.75	5.92	43	14.96	3.35	0.81	84.8	0.418	0.163	negligible
folic acid	53	11.35	7.26	44	9.21	4.10	1.83	84.5	0.071	0.363	small
rheumatoid factor	53	31.40	88.35	44	46.18	146.29	-0.59	67.8	0.559	-0.122	negligible
anti-CCP antibodies	53	1.95	10.89	43	6.48	40.76	-0.71	46.9	0.483	-0.152	negligible
beta2-microglobulin	53	2.01	0.70	44	2.20	1.22	-0.91	66.0	0.364	-0.191	negligible
creatine kinase	53	119.55	85.05	44	118.11	90.04	0.08	89.6	0.936	0.016	negligible
immunoglobulin A	49	2.44	1.12	43	2.32	1.06	0.55	89.4	0.585	0.114	negligible
TSH	53	1.33	1.06	44	1.43	0.90	-0.51	95.0	0.612	-0.103	negligible
fT3	53	4.55	2.39	43	4.18	0.76	1.08	64.5	0.284	0.212	small
fT4	53	13.03	2.98	43	12.15	2.36	1.60	94.0	0.112	0.325	small
anti-TPO antibodies	53	45.06	122.17	43	25.95	50.30	1.04	72.1	0.304	0.205	small
anti-TG antibodies	53	195.30	809.22	43	58.24	178.99	1.20	58.2	0.236	0.234	small
carcinoembryonic antigen	53	1.75	1.06	44	1.85	1.76	-0.33	67.5	0.742	-0.069	negligible
Ca125	46	14.84	9.55	43	17.89	11.64	-1.34	81.4	0.183	-0.286	small
H. pylori antigen	52	50.43	65.88	42	34.86	56.31	1.24	91.7	0.220	0.254	small
anti-AChR antibodies	51	0.31	0.19	44	0.34	0.28	-0.64	74.2	0.526	-0.133	negligible
